# Hospital Expenditure.—The Commissariat

**Published:** 1896-05-02

**Authors:** 


### THE INSTITUTIONAL WORKSHOP.

### HOSPITAL EXPENDITURE.—THE COMMISSARIAT.

### XV.—CONSUMPTION OF MEAT.

The most important item of the food supply is meat. Taking thirteen of the largest London general hospitals together, we find that exactly one-third of their total expenditure on provisions is spent on what is commonly called butcher's meat, as distinguished from bacon, poultry, &c. A comparison of the butchers' bills of various hospitals reveals a curious discrepancy in the cost of this article per head. We have already many times pointed out the fallacy of making any reckoning of cost in hospital finance on the basis of dividing all the money spent among the occupied beds, since the wide variations which result are due to internal arrangements in the hospital for the most part entirely unknown to the public who criticise the summaries presented to them. From inquiries made in different localities, and from a consideration of the diet-sheets of the leading hospitals throughout the country, we have reached the conclusion that the cost of the patient varies but little, though higher in London than in the country. In presenting comparative tables of the cost of meat in the various hospitals, London and provincial, we have, therefore, set aside a fixed amount for the patients' share, and have adopted as the average cost of each patient for this article in London hospitals the estimate of 3d. a head a day, allowing for ½ lb. of meat (as received from the butcher, uncooked, and with bone), at an average of 6d. per lb. This amounts to £4 11s. 3d. a year, and deducting the odd shillings as a set-off against those on milk diet (patients receiving ordinary beef-tea must reckon for this purpose as meat consumers), it is believed that £4 per bed yearly will be found a safe working average for the cost of butcher's meat for the patients. Indeed, a comparison of this estimate with an accurately worked out calculation of the amount spent on butcher's meat for the patients at Guy's in 1893-4 showed the results to be identical, viz., £4 for each patient in the year.

| Hospital. | Daily average of patients. | Number of staff. | Staff—how constituted. | Yearly cost of butcher's meat. | Cost of patients' meat at £4 a head. | Remainder of butcher's bill divided among staff. Pr hd. £ | s. |
|---|---|---|---|---|---|---|---|
| | | | | £ | £ | £ | s. |
| London Hospital... | 656 | 77 | Doctors, household | 4,531 | 2,624 | 24 | 15 |
| Guy's ... ... | 411 | 35 | Household only | 1,876 | 1,644 | 7 | 0 |
| St. Thomas's ... | 365 | 46 | Household only | 1,740 | 1,460 | 6 | 0 |
| St. George's ... | 323 | 183 | Doctors, nurses household | 2,993* | 1,292 | 9 | 6 |
| St. Mary's ... ... | 251 | 122 | ,, ,, | 2,452 | 1,004 | 11 | 17 |
| Middlesex ... ... | 215 | 141 | ,, ,, | 2,954 | 860 | 14 | 17 |
| Seamen's ... ... | 214 | 69 | ,, ,, | 1,885 | 856 | 15 | 0 |
| University... ... | 186 | 54 | Doctors, household | 1,398 | 744 | 12 | 0 |
| Westminster ... | 176 | 48 | Doctors, some nurses, hsehld. | 1,307 | 704 | 12 | 11 |
| Charing Cross ... | 150 | 92 | Doctors, nurses household | 1,679 | 600 | 11 | 3 |
| Royal Free ... | 112 | 70 | ,, ,, | 866 | 443 | 6 | 0 |
| Gt. North. Central | 68 | 38 | ,, ,, | 685† | 272 | 10 | 17 |
| Metropolitan ... | 47 | 50 | ,, ,, | 826 | 188 | 12 | 13 |

* Includes Brand's Essence. † Includes bacon.

As regards officials no estimate of what may be considered a fair average can be given; but it may be noted that at the London Hospital Nursing Home the butcher's bill divided among the inmates (275 nurses and 14 female servants) shows a yearly average per head of £6, or about 4d. a day. Having deducted in the foregoing table at the rate of £4 for each patient, the remainder of the butcher's bill has been divided among the officials, and with every allowance for difference in prices and quality the variations can hardly be called satisfactory.

It is not of course meant to suggest that no more than £4 per head yearly is ever expended on the patient's meat; in some hospitals, the London, for instance, this amount evidently is exceeded. It may be pointed out that an extra 1d. a day on each patient's meat at a hospital the size of the London would make the difference of £1,000 a year in the butcher's bill, and this would reduce the allowance of the staff to £11 15s.

Turning to provincial institutions and adopting a similar estimate of the patients' cost for meat, it becomes at once evident that the yearly average of £4 a head is far too high, the remainder of the bill to be divided among the staff revealing in one instance a minus quantity and in many a sum obviously insufficient. The quarterly return of the Birmingham General Hospital shows an average of 2 lbs. of meat per head consumed among the patients weekly, and in some provincial hospitals where the quantities used yearly are stated in the report, it seems probable that even less than this is used among the patients, the average for all the inmates taken together seldom exceeding 3 lbs. a week; £3 a year, then, or rather under 2d. a day for each patient, is probably for provincial hospitals a more accurate statement, the design being less to hazard conjectures about the patients' cost than to render mutual comparison possible and helpful. In all cases the staff consists of doctors, nurses, and household.

| Hospital. | Daily average of patients. | No. of staff. | Yearly cost of butcher's meat. | Cost of patient's meat at £3 a head. | Remainder of butcher's bill divided among staff. Per hd. £ | s. |
|---|---|---|---|---|---|---|
| | | | £ | £ | £ | s. |
| Birmingham General ... ... | 231 | 125 | 1,457 | 693 | 6 | 2 |
| Birmingham Queen's ... ... | 113 | 55 | 679 | 339 | 6 | 3 |
| Bristol General ... ... ... | 172 | 90 | 1,135 | 516 | 6 | 16 |
| Cambridge (Addenbrooke) ... | 56 | 56 | 684 | 168 | 9 | 0 |
| Hull ... ... ... ... | 160 | 63 | 1,119 | 480 | 10 | 0 |
| Leeds General ... ... ... | 355 | 159 | 2,238 | 1,065 | 7 | 7 |
| Liverpool Royal Infirmary ... | 245 | 75 | 1,283 | 735 | 7 | 6 |
| Norfolk and Norwich ... ... | 134 | 64 | 937 | 402 | 8 | 7 |
| Newcastle-on-Tyne ... ... | 256 | 111 | 1,858 | 768 | 9 | 16 |
| Oxford Radcliffe ... ... | 105 | 69 | 805 | 315 | 7 | 0 |
| Sheffield ... ... ... ... | 178 | 78 | 1,525 | 534 | 12 | 13 |
| Sussex County ... ... ... | 134 | 75 | 737 | 402 | 4 | 14 |
| York County ... ... ... | 103 | 42 | 706 | 309 | 9 | 9 |
| Halifax ... ... ... ... | 92 | 41 | 591 | 276 | 7 | 13 |
| Aberdeen ... ... ... ... | 157 | 87 | 1,029 | 471 | 6 | 9 |
| Ayr County ... ... ... | 54 | 29 | 366 | 162 | 7 | 0 |
| Edinburgh Royal Infirmary ... | 690 | 288 | 4,555 | 2,070 | 8 | 12 |
| Glasgow Royal Infirmary ... | 543 | 236 | 3,435 | 1,629 | 7 | 13 |
| Glasgow Western Infirmary ... | 388 | 165 | 2,333 | 1,164 | 7 | 1 |

Many of the variations observable in the above table will be found to be due to difference in prices according to locality. The variations, however, are less marked than among the London hospitals, where one might expect a more even standard of prices, and the range from £4 14s. a year for each member of the staff at the Sussex County Hospital to £12 13s. at Sheffield is a difference more easily accounted for than that between the Royal Free at £6 a head and the London Hospital at £24 15s.

